# Supplementary material for: “My father insisted that I have the baby but not in his house”: Adolescent pregnancy, social exclusion and (dis)empowerment of girls in an urban informal settlement in Kenya
Source: PLOS Glob Public Health. 2024 Sep 26;4(9):e0003742. doi: 10.1371/journal.pgph.0003742 (PMC11426473; doi:10.1371/journal.pgph.0003742)
Supplement: S1 Text — These are the interview guides used to conduct interviews with participants. The guides were designed to explore the lived experiences of pregnant and parenting adolescents. Separate guides were developed for different respondent categories, including adolescents, teachers, policymakers, parents/guardians, chiefs, village heads, sub-county officers in charge of children, and healthcare providers. Each guide includes sections with open-ended questions to elicit detailed responses about personal challenges, perceptions, and experiences related to adolescent pregnancy and parenting. (DOCX) [file pgph.0003742.s001.docx]

**INTERVIEW GUIDES**

We are conducting this study to understand the lived experiences of pregnant and parenting adolescents. No personal identifying information will be collected. Any data provided will be treated in adherence to the highest ethical standards for conducting human subjects research. Privacy, anonymity, and confidentiality will be respected.

**QUESTIONS TO ADOLESCENT PARENT**

**Background information**

Tell me about yourself (I would like to know your age, marital/cohabiting status, who you live with, the age you became pregnant, whether you were still in school when it happened, the number of children you have, and if you are still in school, or working, or just at home)

**Circumstances surrounding adolescent pregnancy**

1. Tell me, how and when did you find out you were pregnant? Who got you pregnant?
2. Were you planning to be pregnant when you got pregnant?
   1. If not, what was the main reason you would say is responsible for you becoming pregnant? Probe for access to contraceptive information and services as well as sexual violence, probe for who the partner is, including age
3. What was your reaction to you getting pregnant?
4. How did you inform your partner, and what was his reaction to your pregnancy? How did his reaction make you feel? Did he take responsibility for the pregnancy?
5. How and when did you inform your parents? How did your parents react? Probe separately for father and mother reactions. How did their reactions make you feel?

**Community attitudes to adolescent pregnancy**

1. What is the attitude of your peers, church, and the community towards you becoming pregnant? Positive or negative
2. Why do you think they act that way?
3. How did their reactions make you feel?
4. Have their attitudes changed since you gave birth? If yes, how?

**Adolescent pregnancy and experiences in health care facilities (pathways to prenatal care and experiences with care)**

1. Did you receive antenatal care when you were pregnant? Yes, No.
   1. If yes, how far into the pregnancy did you begin receiving antenatal care? Probe: if not in the 1st trimester (3 months), probe for why the delay in seeking care.
   2. How would you describe your experience at the clinic? Friendly, excellent?
   3. If no, why not? Were there barriers to accessing care? Explain. Probe for attitudes of health workers, the quality of care, whether they were exposed to stigma, verbal abuse, etc. Probe for overall satisfaction with care received.
2. Where did you deliver your child and why? Did you receive postnatal care?
3. Did you take your child to a health facility for immunization regularly? Yes, No.
   1. If no, why not? What are the barriers preventing you?
4. Where do you take your child when s/he is unwell? Why?
5. How would you describe the impact of your pregnancy on your physical and mental health?
6. [Parenting adolescent] What are the main challenges that you faced during your pregnancy? [Pregnant adolescent] What are the main challenges that you are currently facing as a pregnant adolescent?

**Childcare and support (skip for those still pregnant)**

1. On a day-to-day basis, who takes care of your child?
2. Who supports you in caring for the child? What kind of support do they provide? Explain.
   1. The father of the child?
   2. Your family?
   3. Friends/peers? Probe for specifics
3. Is their support, particularly in terms of providing resources to care for your baby, adequate?
4. What are the main challenges you are facing in bringing up this child? Probe for specifics and coping strategies for each challenge

**Impact of adolescent pregnancy on education attainment**

1. What happened with your education when you became pregnant?
2. [If dropped out of school when they got pregnant] Do you intend to continue your education or get further training?
3. Do you know about the school re-entry policy?
4. How do you think pregnant and young mothers can be best supported within the educational system?
5. Have you returned to school since childbirth?
6. [If yes] What was the main factor that encouraged you to return to school? What are the main challenges you currently face in school?
7. [If no] What is the main reason you have not returned to school? Do you intend to return to school in the future? What do you need to help you return to school? What are your fears about returning to school?
8. What could be done to support adolescent mothers’ education and facilitate school re-entry?

**Impact of adolescent pregnancy on livelihoods**

1. What is your current source of income? Is your current source of income enough to provide for you and your child?
2. [If no] How do you cope?
3. What would you need in order to be able to provide adequately for yourself and your child?
4. What plans do you have for the future yourself? For your child?

**INTERVIEWS WITH TEACHERS AND POLICYMAKERS**

1. In your school, what happens to a teenage girl when she becomes pregnant?
2. What is the school’s official policy on teenage pregnancy? Probe: Does your school allow back students who have delivered and are willing to continue with their studies? How many come back?
3. What are the policy guidelines from the Ministry of Education on handling situations of pregnant schoolgirls?
4. What do you do to prevent teenage pregnancy in school? Probe: Are there prevention measures specific to the COVID-19 pandemic?
5. In your opinion, what can schools do to prevent teenage pregnancy?
6. What do you currently do to support teen mothers returning to school after childbirth?
7. What can schools do to support teenage mothers willing to return and complete their studies?
8. What may prevent teen mothers to go back return to school?
9. What challenges do they experience when they come back to school?
10. Any challenges at home that might prevent them from going back to school?
11. Are there COVID-19-related challenges that may prevent them from returning to school?
12. What could be done to support teenage mothers in school?
13. What could be done to facilitate school re-entry of teenage mothers?

**INTERVIEWS WITH GUARDIANS**

Tell me about yourself

1. How did you find out your child was pregnant?
2. What were your immediate reactions?
3. What and who do you blame for her becoming pregnant?
4. How have you been supporting her since you found out she was pregnant?
5. What were the main health challenges she faced? Probe for how they coped/tried to solve the health challenges.
6. What were the main economic challenges she faced? Probe for how they coped/tried to solve the economic challenges.
7. How has pregnancy affected her education chances? Probe for how they coped/tried to solve the education challenges.
8. How could you support her to return to school?
9. What can be done to prevent teenage pregnancy?
10. What could be done to support teenage mothers in school?
11. What could be done to facilitate school re-entry of teenage mothers?

**INTERVIEWS WITH PARENTING ADOLESCENT BOYS**

Tell me about yourself

1. Did you plan to get your girlfriend pregnant?
2. What do you consider to be the main reasons for the pregnancy? Probe for knowledge of and access to contraceptives.
3. What was your reaction when you became aware that your girlfriend was pregnant?
4. How did your parents react?
5. In what ways has the pregnancy affected you? (mental health (worried, sadness, anxiety, fear), education (school dropout) and or Stigma, exclusion, rejection, or discrimination)
6. What have you done to support your girlfriend since becoming pregnant?
7. How do you now feel about pregnancy and becoming a father?
8. What does fatherhood mean to you?
9. What do you find challenging?
10. What do you find rewarding?
11. Has your parents’ attitude changed since the birth of your baby? If so, in what ways?
12. How has the pregnancy affected your relationship with your girlfriend?
13. What are your plans and aspirations going forward? Probe for school reentry if not in school currently.
14. What can be done to support teenage fathers and mothers?

**INTERVIEWS WITH CHIEFS, VILLAGE HEADS, AND SUB-COUNTY OFFICERS IN CHARGE OF CHILDREN**

Tell me about yourself

- - - 1. How common is teenage pregnancy in your community?
      2. What are the main causes of adolescent pregnancy in your community?
      3. What is your experience in handling cases of adolescent pregnancy?
      4. What does your office do in cases of adolescent pregnancy?
      5. How do you resolve paternity dispute issues?
      6. How do you participate in the prevention of teenage pregnancy? What do you do to prevent teenage pregnancy?
      7. What adolescent sexual and reproductive health programs are being implemented in your community? Who is implementing these programs? What services are they providing? Are there programs helping adolescent mothers or pregnant girls? What is the nature of the help they provide for these girls?
      8. In your opinion, what happens to girls when they become pregnant? What are the challenges they face?
      9. How are pregnant and parenting adolescents treated in your community?
      10. How does pregnancy affect their education?
      11. Why do pregnant and parenting girls rarely return to school after delivery?
      12. What more can be done to support adolescent mothers and pregnant adolescents?
      13. What more can be done to end adolescent childbearing in your community?
      14. What can the community leadership do to facilitate school reentry of out-school-adolescents?

**INTERVIEWS WITH HEALTHCARE PROVIDERS**

Tell me about yourself

1. Please tell me about a case of an adolescent client (10-19 years) you have provided service for in the recent past.
2. When did you interact with the client and what services did you provide?
3. What made you remember this case/client? what stood out about the client?
4. How old were they?
5. How unique was the client/case compared to other clients?
6. Can you tell me a little about what you do in relation to the provision of ANC for adolescents?
7. Typically, what trimester do they present for antenatal care?
8. What are the reasons some present late for antenatal care?
9. What services are offered them during ANC? (HIV testing, psychosocial counseling/mental health support, contraceptive counseling, etc. )
10. What is your understanding of patient-provider communication?
11. Can you briefly tell me about how you typically talk to girls about services (Probe: do you give them information on reasons for medications and procedures; are adolescents confident to ask you questions and be answered).
12. What would you say about how you relate with adolescent clients? Probe about attitudes of other staff at the facility such as doctors, nurses, and clinical officers. (Probe specifically for experiences of stigma, discrimination, verbal abuse, and physical abuse)
13. Are these services offered for free? Are the services accessible all the time?

(Probe: How do young girls pay for the services if they are not free?)

1. Are these services readily available for adolescent girls?
2. How do you perceive young women (10-19 years) seeking ANC care?
3. Beyond offering services, how else do you support young women to access antenatal care? Probe for creating awareness within the community, offering advice to young women
4. What are the key barriers young women experience in accessing ANC/PNC services?
5. How do you ensure the privacy/identity protection & confidentiality of GIRLS who come for ANC? [Probe for: at the waiting bay, examination rooms; patient records/data/information]
6. What are some of the supportive services or structures available that you provide adolescent clients after offering the services?
7. Do you do patient follow up? How do you do it?
8. Do you offer post-partum contraception? What type of contraception is offered? (long term vs. short term) Why? What are the available methods? Do you experience commodity stockout?
9. To what extent do young mothers leave clinics with contraceptives after delivery?
10. What methods do they typically leave with?
11. Are young mothers counseled on contraceptive methods? What information is typically provided during contraceptive counseling?
12. What are some of the reasons some do not leave with contraceptives?
