# Supplementary material for: “My father insisted that I have the baby but not in his house”: Adolescent pregnancy, social exclusion and (dis)empowerment of girls in an urban informal settlement in Kenya
Source: PLOS Glob Public Health. 2024 Sep 26;4(9):e0003742. doi: 10.1371/journal.pgph.0003742 (PMC11426473; doi:10.1371/journal.pgph.0003742)
Supplement: Attachment — Submitted filename: Response to reviewers.docx [file pgph.0003742.s002.docx]

Beryl Nyatuga Machoka

berylninanyatuga@gmail.com

Postal Adress: 10787-00100

Nairobi, Kenya.

July 30,2024

The Editor

PLOS Global Public Health

Dear Editor,

**Re: Point by Point Response to the Revision Request for Manuscript PGPH-D-24-00309:
“My father insisted that I have the baby but not in his house”: Adolescent pregnancy, social exclusion and (dis)empowerment of girls in an urban informal settlement in Kenya.**

I am writing to formally share the revised version of our manuscript titled “My father insisted that I have the baby but not in his house”: Adolescent pregnancy, social exclusion and (dis)empowerment of girls in an urban informal settlement in Kenya.

We thank the reviewers for their comments. We have addressed them accordingly and provided a point-to-point response, as shown below.

Reviewer #1: Thank you for your interesting manuscript. This paper explores the important question of the interrelation between early pregnancy and processes of social exclusion. Although I find the approach interesting, I have some major concerns.

**Introduction**

• In the introduction different aims of the study are formulated (p. 1, p. 5). No clear research question is formulated

Response:

This information has been added to the paper.

Thus, this study attempts to answer two questions: 1) how does early pregnancy shape social exclusion experiences of pregnant and parenting girls and 2) what implications do social exclusion have for pregnant and parenting girls’ education and socioeconomic empowerment?

• The importance of parents is not sufficiently emphasized

Response:

The role of parents is incorporated primarily to provide context for the social exclusion experiences of pregnant and parenting adolescent girls. This includes their socio-economic status, their reactions or attitudes toward the girls, their involvement in decision-making processes such as school reentry, and the girls' access to resources.

The role of parents is discussed under the “Social Exclusion, Adolescent Pregnancy, and Girls’ Disempowerment” as follows:

“With shrinking opportunities, more and more parents are excluded from decent work with sufficient income. We argue that the social exclusion of parents of adolescent girls from decent jobs that earn living wages enough to escape the poverty trap leads girls to engage in survival sex resulting in early and unintended pregnancy. The exclusion of willing and able parents from decent living and meaningfully paying jobs is the largest pull to poverty(42,43). Parents' lack of jobs and precarious jobs significantly limit their ability to provide for their children. Likewise, precarious work means parents work long hours in low-paying jobs and significantly spend time away from their children. This limits their ability to supervise their children and deter them from engaging in survival sex. Studies have shown that lack of parental support and supervision are associated with transactional sex and adolescent pregnancy (16). Adolescent girls born to middle-class to rich parents rarely become pregnant early and are largely shielded from the negative consequences of early childbearing (44).”

• The argument is very abbreviated to economic factors for early pregnancy. Other factors are certainly relevant here (cf. e.g. results from resilience research), which are not taken into account here.

Response:

Our argument relates to how social exclusion leads to adolescent pregnancy and childbearing. We explored three ways this happens, first is through material deprivation leading girls to exchange sex for money, second is through denial of access to SRH information to equip them to prevent pregnancy and third is through active exclusion from the use of contraceptives. We lay out this in our argument. While incorporating resilience research would have been valuable, this would have expanded the scope beyond the focus of this study to analyzing why girls are getting pregnant or not getting pregnant.

• The term socioeconomic well-being should be defined.

Response:

The definition of socio-economic well-being has been added to the paper.

Socioeconomic well-being refers to the overall economic and social status of individuals or households, encompassing factors such as income, education, employment, access to basic services, and quality of life.

• You argue that girls interchange basic needs for sex. What do you exactly mean by basic need?

Response:

"Basic needs" refer to essential requirements for survival and well-being, including food, shelter, clothing, money, sanitary pads, and personal effects. This information has been added to the manuscript.

• In my point of view, it would be helpful to clarify active exclusion for marginalised communities (p. 5). The argumentation in this section is not entirely conclusive.

Response:

We have revised the section to better illustrate how policies restricting adolescents' access to contraceptives exacerbate their exclusion in marginalized communities. This is especially true in low-income areas where healthcare affordability and infrastructure are significant challenges. See the section below:

“Second, Amartya Sen distinguishes between passive and active exclusion. He theorizes that active exclusion results from deliberate actions, such as policies intentionally denying young people access to comprehensive sexuality education CSE or contraceptives. Research has shown that young people value privacy when seeking sexual and reproductive healthcare services (45). In low-income areas, adolescents often face barriers such inability to afford healthcare and inadequate healthcare infrastructure (46). Therefore, enacting policies that restrict their access to contraceptives can further exacerbate their exclusion and increase their vulnerability to risks associated with early unintended pregnancies. Also, requiring parental consent before sexual and reproductive health services are provided to adolescents who seek these services could mean that adolescents forfeit accessing services altogether. The implication of excluding adolescents from critical preventive information and sexual and reproductive health information and services is that they are disempowered in preventing early and unintended pregnancy. Also, punitive policy that expressly prohibits pregnant and parenting girls from formal schooling, such as in Tanzania and Sierra Leone—although now reversed (47)—is another example of active exclusion.”

• Can you please elaborate on the theoretical positioning of both the social exclusion framework and intersectionality theory (and in particular the connection between the two theoretical approaches)?

Response:

Amartya Sen's social exclusion framework serves as the theoretical underpinning of our study. We incorporate intersectionality to explain that various factors contribute to social exclusion. We added that: Intersectionality is an analytical framework in sociology that explores the intersections of different social and political identities, leading to distinct experiences of discrimination and privilege. It emphasizes that various aspects of identity are interconnected intricately, leading to advantages or disadvantages, and helping to understand the complexity of human experience.

• Please give more information about the situation of adolescent pregnancy in Kenya (especially focusing on mechanisms of social exclusion).

Response:

This is the goal of this paper, and we believe we did that in the results and discussion sections. In the introduction, we added the following:

In Kenya where 15% of adolescent girls start childbearing before the age of 19, research reveals that social stigma, including avoidance, ridicule, and mistreatment, against adolescent pregnancy forces girls to hide or relocate to new residences (16,18). These pregnancy-related stigma result in poor health outcomes, low educational attainment, and economic inactivity that expose girls to dependence and loss of individual agency in society (16,18,25). Of particular concern are urban slum settlements such as Korogocho in Nairobi, which are characterized by dense populations, inadequate infrastructure, and limited access to essential services (16). Compared to their counterparts in non-slum areas, girls in urban Kenyan slums face heightened vulnerabilities to early pregnancy due to high levels of sexual violence, insecurity, socio-economic disparities, limited access to education, and contraceptive and reproductive health information (16,26,27).

**Methods Section**

• You describe that the results come from a mixed-method study. What is the connection between qualitative and quantitative data? And why are only qualitative data reported here?

The data came from a larger study with broader objectives of understanding the lived experiences of pregnant and parenting girls. Only the qualitative data are relevant to the objective of this study.
• I would suggest that you use the COREQ guidelines to report on the qualitative methods. There are some important issues missing (e.g. length of interviews, data saturation, data validation).

Response:

We have revised this section to incorporate important aspects recommended by the COREQ guidelines, such as the gender of the research assistants, data saturation, and data validation processes. The manuscript contains information on the duration of the interviews.

• Can you please add the interview guide and the coding tree as a supplemental file?

Response:

The interview guide and coding tree have been added as supplemental files supplemental files.

**Results**

• The structure of the results section has been chosen appropriately. I would go into more detail on the conditioning factors for social exclusion, precisely because different groups (cf. p. 6) were defined. I suppose that the experiences of early pregnancy are certainly very different between the subgroups. This would also help to broaden the intersectional view (which was stated in the introduction).

Response:

In response to the suggestion to broaden the intersectional view by considering varying experiences within this homogenous group. Even though pregnant and parenting girls in a low-income informal settlement are homogenous in terms of their vulnerabilities, there are a few areas of differences in their experiences and we have highlighted this when the data allow.

• There are only a few results of the second group of participants. Are there differences and/or similarities between adolescents and adults?

Response:

We have integrated insights from the second group of participants, particularly parents, which were previously underrepresented. Comparing adolescents and adults in the study reveals similarities in their reports. Both groups emphasize poverty and the lack of comprehensive sexuality education as primary factors influencing adolescents' early entry into sexual relationships and consequent pregnancies. Additionally, they agree on social exclusion experiences through community stigmatization, parental decisions to withhold support for school re-entry, as well as adolescents' reluctance to continue with education due to societal stigma.

The differences noted are that the adolescents emphasize the immediate impacts of pregnancy on their social lives, such as isolation from peers and school-related challenges like stigma and dropout rates. They also tend to focus on personal hardships and emotional responses to parental reactions, including feelings of abandonment or harsh discipline. In contrast, adults, emphasize broader socio-economic factors influencing the girls’ pregnancies, such as financial instability and the challenges of balancing work responsibilities with parenting duties.

• The role of parents is only presented from the girls’ perspective. How do the parents see their own role?

Response:

We have incorporated the perspective of parents into the analysis. Parents acknowledged financial challenges, relying on low-wage jobs and struggling to provide essentials like food and school fees. They expressed concerns about how economic stress reduced their ability to supervise their daughters, potentially contributing to early pregnancies. Parents also noted limited access to sexuality education as a factor influencing their daughters' understanding of contraception. Parents also reported having harsh reactions such as avoidance and withdrawal of educational support upon their daughters’ pregnancy.

• The last quote is not sufficiently integrated into the text

Response: We have now integrated the last quote into the text.

**Discussion**

• The interrelated factors mentioned (p. 13) are not sufficiently emphasized in the results section. In my view, as I said, it makes sense to compare different groups and their experiences in order to illustrate intersections between conditions of social exclusion.

Response:

Pregnant and parenting in low-income informal settlements are similar in terms of their characteristics. We highlighted where their experiences differ. We have also indicated that future study should explore how different groups of pregnant and parenting adolescents, including those living rural areas and urban rich areas experience social exclusion.

• The references to other studies are worked out. But what is really new about this study? What were the surprising findings?

Response: This information has been added to the discussion section.

An intriguing observation from our research is that the passive social exclusion of parents from earning living wages, coupled with their unstable employment conditions marked by prolonged work hours away from home, has led to an increased vulnerability of their daughters to early and unintended pregnancies. Due to parents working extended hours away from home and earning minimal wages, their daughters frequently remain unattended and unsupervised, which can leave them vulnerable to negative influences

While this study aligns with previous research on the economic vulnerabilities and educational challenges faced by adolescent girls, it offers new insights by highlighting the compounded effect of both passive and active social exclusion that begins even before conception. Unlike previous research, it uncovers how material deprivation and inadequate comprehensive sexuality education not only lead to early pregnancy but also sustain a cycle of marginalization and exclusion. Another surprising finding is the extent to which societal stigma delays antenatal care and increases health risks for both mother and child. Additionally, unlike some contexts where adolescent mothers receive substantial support to return to school, this study reveals that in low-income settings, the lack of financial and social support significantly hinders their educational and economic recovery post-pregnancy.

• The discussion point about the anti-right movement remains very vague. What role did that play in the interviews?

Response:

We have limited the sentence on the anti-right movement to only one sentence and kept the focus on how lack of access to accurate sexual and reproductive health information hinders girls from preventing unintended pregnancy in line with the study findings.

• Regarding policy implications: It would be helpful to concretise strategies to improve the girls’ situation.

Response:

The policy implications have been refined to include concrete strategies aimed at improving the conditions for adolescent girls facing challenges related to early pregnancy and social exclusion.

• Regarding study strengths and limitations: please check the COREQ-guidelines

Response:

We have revised the section in line with COREQ guidelines. We have clarified, and emphasized the relevance of our findings to the study context, and acknowledged potential biases that may impact our results.

**Minor concerns**

• There are minor redundancies in the introductory section.

Response: we have revised the introduction section.

Reviewer #2: Thank you for the opportunity to review this paper. I must congratulate the authors for the well-written manuscript. Nevertheless, I believe there are still some points for improvement.

**Abstract**

1. The methods section of the abstract should indicate the study design.

Response: We have incorporated the study design into the abstract.

2. Keywords: I would suggest replacing wellbeing with 'Reproductive Health', and delete 'health'.

Response: Done

**Introduction and background**

3. While the introduction is straightforward, it is quite limiting. The authors have to help readers understand how rife adolescent pregnancy is in Kenya and particularly in slum settlements. Then, they must zoom in to the differences in rural and urban slums so that readers can appreciate why the focus is on urban slum settlement.

Response:

Additional information has been provided on the prevalence and contextual factors contributing to adolescent pregnancy in Kenya.

4. The authors must dedicate a section to discuss Sen's social exclusion framework. Let us understand what it is, its assumptions and how it fit this study.

Response: We had already included a section that discusses Sen's social exclusion framework and its application to our study. We have enhanced this section with additional details to clarify the framework’s assumptions.

**Methods**

5. Why was qualitative explanatory design used? You should remember that your methodology is also literature to other researchers.

Response: This information has been added to the manuscript.

We adopted a qualitative explanatory design (55), to gain insight into participants’ experiences of social exclusion leading to their early pregnancy and resulting from their becoming pregnant. The analytical goal of comprehending the effects of social exclusion on adolescent pregnancy, as well as why early childbearing might intensify social exclusion, makes a qualitative explanatory design the most suitable methodology for this study.

6. How was the study site 'Korogocho' selected? Is that the only urban slum? We need to understand the rationale behind this selection.

Response: This information has been added to the manuscript.

The selection of Korogocho was intentional, partly influenced by its high prevalence of adolescent pregnancy and because it has been the site of our Nairobi Urban Health and Demographic Surveillance System (NUHDSS) for over two decades. Korogocho's demographic and socio-economic characteristics provide context for examining the intersection of poverty, social exclusion, and early pregnancy among adolescents.

7. "Through a community-based organization in the study setting..." Which community-based organization? Does any of the authors work with this organization? If yes, how was reflexivity ensured?

Response: This information has been added to the manuscript.

We collaborated with an independent community-based youth empowerment organization (CBO) focused on addressing the vulnerabilities of young women and adolescents in the study area to recruit the participants. None of the authors worked in the CBO. Through this partnership, outreach staff from the CBO identified and purposively sampled adolescent girls and adults who met the inclusion criteria.

8. How did you select the second and third group of participants?

Response: This information has been added to the manuscript.

Staff from the CBO utilized physical tracing in the community, referrals, and existing records to identify and invite the first and second group of participants to take part in the study. The inclusion criteria for this group required individuals to be legal guardians or parents of pregnant or parenting adolescent girls residing in the study site.

The third group consisted of key informants such as teachers, policymakers, community leaders, health workers, and NGO representatives. To identify and recruit them, the CBO conducted physical searches throughout the community, cross-referenced their existing records, and leveraged contacts within their professional network. The inclusion criteria required that they either lived or worked in the study site and had direct interactions with the community’s pregnant and parenting girls and their parents.

9. It is not enough to say 'purposively sampled'. Practically, what did you do? This is vital to validate your methodology and inform other researchers.

Response: This information has been added to the manuscript.

10. At what point did you stop the interviews? If it was by saturation, then what was the stopping criteria?

Response:

In terms of saturation, our sample size was motivated more by the need for diverse views and triangulation of data, therefore this guided our sample size more than the need for saturation. We grappled with uncertainty and acknowledged that meaning arises from the interpretation of data. Also, decisions regarding 'how many' data items are needed, and when to cease data collection, are inherently subjective and cannot be wholly predetermined before analysis. As such, we conducted in-depth interviews with girls (n=22), parents (n=10), and key informant interviews with teachers (n=4), policymakers (n=3), community leaders (n=6) non-governmental organization representatives (n=2), and health workers (n=4) to be able to triangulate information. Adequate sample sizes to reach saturation in interviews remain a controversy (See Sebele-Mpofu, F. Y. (2020). Saturation controversy in qualitative research: Complexities and underlying assumptions. A literature review. Cogent Social Sciences, 6(1), 1838706; Braun, V., & Clarke, V. (2024). How do you solve a problem like COREQ? A critique of Consolidated Criteria for Reporting Qualitative Research. Methods in Psychology, 100155. and Braun, V., & Clarke, V. (2021). To saturate or not to saturate? Questioning data saturation as a useful concept for thematic analysis and sample-size rationales. Qualitative research in sport, exercise and health, 13(2), 201-216.). A review of the literature suggests between 9 to 17 interviews and 4-8 focus group discussions are sufficient to reach saturation (See Hennink, M., & Kaiser, B. N. (2022). Sample sizes for saturation in qualitative research: A systematic review of empirical tests. Social science & medicine, 292, 114523.).

11. The authors must specify what they mean by, "Four well-trained research assistants conducted face-to-face interviews". What was their educational level and experience in conducting such research?

Response:

Information on the research assistants' education level is included in the positionality statement. All four research assistants were bilingual, and two had personal experience with adolescent pregnancy. They were university graduates and had prior experience in qualitative data collection within the study setting, which helped them relate easily with the participants.

12. Was there any training for the research assistants? If yes, how long and what did it cover?

Response:

Yes, the research assistants received training for 5 days. Comprehensive details regarding this are provided in the data collection section.

13. At what point what inductive analysis done and what point for deductive analysis?

Response:

This information has been added to the manuscript. Inductive analysis was conducted initially to allow themes to naturally emerge from the data. Once key themes and patterns were identified, deductive analysis was applied to further examine these themes within the context of Amartya Sen’s social exclusion theoretical framework.

14. What qualitative research framework informed the thematic analysis?

Response:

The data were analyzed using thematic analysis drawing upon Amartya Sen's framework of social exclusion, and grounded in interpretive phenomenological analysis.

15. How did you ensure rigor and trustworthiness?

Response:

Throughout the study, we maintained rigor and trustworthiness to ensure the integrity and utility of the findings. We were meticulous, transparent, and critical, continually scrutinizing our methods and results while seeking alternative explanations. To verify the completeness and accuracy of the data, we compared transcript samples with the audio recordings. We also triangulated the perspectives of various study participants and examined both positive and negative cases. Utilizing NVivo software for data analysis, audio recording, and transcription provided a comprehensive audit trail for the data collection and analysis processes. We abided by the standards for conducting human subject research. Research assistants underwent training to uphold rigorous ethical standards throughout the data collection process. Our participant recruitment strategies, which included physical tracing, referrals, and leveraging existing records ensured we had a diverse and representative sample that encompassed adolescent girls, parents/legal guardians, and key informants. Our thematic analysis, which was guided by Amartya Sen’s social exclusion theory, helped to systematically explore themes concerning adolescent pregnancy and social dynamics. We also validated our findings by actively seeking participant feedback and engaging in peer discussions to enhance the reliability of our conclusions. Additionally, ethical protocols, such as transparent consenting processes and participant compensation, were followed to uphold the integrity of the study. The research team’s reflexivity and cultural sensitivity were also integral in minimizing biases and ensuring an authentic portrayal of participant viewpoints throughout the study.

**Findings and discussion**

16. I entreat the authors to develop a diagram showing how the themes and sub-themes fit into Sen's social exclusion framework.

Response:

We have incorporated a visual representation that maps out how the themes identified in our study fit within Sen's conceptual framework of social exclusion. This diagram has been uploaded as a supplementary file.

17. As indicated, providing details of Sen's social exclusion framework would help a lot to better situate the study's findings.

Response: We have expanded on Sen's social exclusion framework and included a diagram as a supplementary file to show how our themes relate to it.

18. "By distilling and synthesizing diverse views of key stakeholders, we provide a detailed analysis of social exclusion associated with adolescent pregnancy". How is this a strength of the study? Is it not what your study intended to achieve?

Response: We have reflected on the methodology and findings to improve the strengths and limitations section.

19. Reflect on the methodology used in this study to beef up the strengths and limitations section.

Response: We have reflected on the methodology and findings to improve the strengths and limitations section.

Regards,

Beryl Machoka
